# Lesion Detection and Analysis Using Optical Imaging

**DOI:** 10.3390/diagnostics13091565

**Published:** 2023-04-27

**Authors:** Viktor Dremin

**Affiliations:** 1 Research & Development Center of Biomedical Photonics, Orel State University, 302026 Orel, Russia; v.dremin1@aston.ac.uk; 2 College of Engineering and Physical Sciences, Aston University, Birmingham B4 7ET, UK

The biomedical application of optical spectroscopy and imaging is currently an active, developing area of research, supported by recent technical progress in the development of light sources and detectors. Furthermore, the development of portable and low-cost optical imaging systems shows strong potential for the implementation of these technologies in everyday life and the daily practice of clinicians. In recent years, many studies have demonstrated that the use of modern optical imaging methods in conjunction with a priori data, as well as advanced data mining approaches, can significantly improve the quality of the medical diagnostic services provided [1,2].

The probing radiation of optical diagnostic methods is scattered due to random spatial variations in tissue morphology. Detection of diffuse reflected or transmitted light can provide information on the scattering and absorbing components of biological tissue. Changes in tissue morphology, including hyperplasia, collagen degradation in the extracellular matrix, and an increase in the nuclear–cytoplasmic ratio associated with the progression of various diseases, can affect the scattering signals. In addition, changes in hemoglobin-absorbing properties can assist with angiogenesis processes, the presence of tissue hypoxia and ischemia, etc. At the same time, incident radiation can excite biological tissue molecules, causing fluorescent radiation. Most endogenous fluorophores are associated with the morphology of the tissue or with various metabolic processes responsible for the functional state of the tissue. Mitochondrial function is an important parameter of tissue viability. According to indicators of respiratory chain activity, it is possible to predict cell death, diagnose tissue ischemia, or, on the contrary, talk about its malignant activity. Additionally, the dynamic change of scattering centers of biological tissues probed by laser radiation leads to the formation of speckle fields, the study of which makes it possible to obtain perfusion (blood flow) maps in various anatomical areas. Thus, biophotonic methods can provide unique opportunities for structural and functional analysis of biological tissues, as well as for early and non-invasive diagnosis and monitoring of the effectiveness of therapy in various diseases [1].

Biophotonics has broad prospects for development, since relatively cheap optical technologies allow images to be obtained and human tissues and organs to be influenced in real time with micron resolution and without the use of ionizing radiation. These technologies have found numerous applications in scientific research and clinical practice: dynamic light scattering methods (laser Doppler flowmetry [3,4], laser speckle contrast imaging [5,6], diffusing wave spectroscopy [7]); diffuse reflectance spectroscopy and visualization, including hyperspectral imaging [8,9]; fluorescence spectroscopy and visualization including lifetime measurements [10,11]; polarimetry [12,13]; videocapillaroscopy [14,15]; optical coherence tomography [16]; THz spectroscopy [17]; various microscopy techniques [18], etc. Modern laboratory and clinical studies show that the use of these instrumental approaches based on the registration of optical irradiation makes it possible to study various diseases from the cellular to the organismal level. Many of the mentioned methods have already reliably occupied their niches in everyday biomedical practice, such as pulse oximetry, optical coherence tomography, indocyanine green fluorescent imaging, and various microscopy realizations. However, further improvements in various optical technologies and their application methodology are still required prior to their large-scale implementation.

This Special Issue is one of many small steps on this long and fascinating journey. It highlights the advantages and unique features of using optical diagnostic technologies for the detection and analysis of lesions. The articles discuss the development and application of optical methods, expanding the current diagnostic capabilities of various diseases, and other aspects of spectroscopy and imaging in biology and clinical practice.

In a case study [19], Y. Goh et al. demonstrated the potential of photoacoustic imaging (PA) as a supplement to ultrasound (US) to increase the reliability of lipoma diagnosis. In this case, the authors showed that, using PA, it is possible to study the biochemical characteristics of fat necrosis. PA can identify the “mass-like” hypoechoic areas on US images as fat-containing, rather than fat-replacing. PA provided information on liquefied necrotic fat from cystic degeneration and fibrosis around the cavity. Therefore, the new biochemical information on fat and collagen content provided by PA can help to recognize ambiguous US results and increase confidence in the diagnosis of fat necrosis.

Several studies in this Special Issue concentrate on the application of Raman spectroscopy in combination with other techniques. Y. Khristoforova et al. combined patient data with Raman and autofluorescence spectral characteristics to better identify skin tumors (melanoma, basal cell carcinoma, squamous cell carcinoma and different benign tumors) [20]. The spectral characteristics of the tumors and patient data were used to construct classifiers based on projection on latent structures and discriminant analysis. Gender, age, and tumor location were found to be significant to the classification of malignant versus benign neoplasms. S. Al-Shareefi et al. applied Raman spectroscopy in dentistry and demonstrated that this method can detect changes in the content of minerals and the collagen matrix in various carious areas [21]. The caries-infected dentin showed a low phosphate peak and higher amide I, III and C-H bond peaks. The amide peaks (I, III) varied in occlusal lesions, as opposed to proximal. Additionally, there was a significant correlation between the mineral:matrix peak ratio and the equivalent Vickers microhardness number within carious lesions.

J. Sachs et al. evaluated the possible impact of dual-energy computed tomography (DECT) on cataract formation [22]. The authors studied the attenuation of X-ray radiation via the crystalline lens, considering clinical and demographic data. The authors concluded that the lens of women and people of color had a higher attenuation during DECT, which may indicate a higher density or increased calcium concentration and a high probability of cataract formation.

P. Glazkova et al. studied the possibilities of incoherent optical fluctuation flowmetry (IOFF) to analyze the perfusion of foot tissues in patients with diabetes mellitus [23]. Perfusion in foot tissues was also assessed using transcutaneous oxygen pressure measurements (TcPO_2_) as a reference standard. The high correlation coefficients were shown between the measurements of the perfusion parameters using IOFF and TcPO_2_. This study demonstrates that the IOFF method allows the identification of patients with a critical decrease in TcPO_2_ < 20 mmHg with sensitivity and specificity of 85.7% and 90.0%, respectively.

The work of E. Zharkikh et al. focuses on studying microcirculation features in patients who underwent COVID-19 using novel wearable laser Doppler flowmetry (LDF) devices [24]. In patients undergoing rehabilitation after contracting COVID-19, a decrease in skin perfusion and changes in the amplitude–frequency behavior of the LDF signal were found. The data obtained confirmed the presence of microcirculatory dysfunction for a long period after recovery from COVID-19.

In a study by E. Cinotti et al., the use of line-field confocal optical coherence tomography (LC-OCT) for the diagnosis of skin cancer was demonstrated [25]. LC-OCT is a new non-invasive imaging technology that combines the benefits of optical coherence tomography and reflectance confocal microscopy in terms of spatial resolution, penetration depth and image orientation, overcoming their corresponding disadvantages and limitations. Considering different types of skin malignant tumors (basal cell carcinoma, squamous cell carcinoma, melanoma), a statistically significant increase in specificity was obtained from 0.73 for dermoscopy to 0.87 for LC-OCT. At the same time, the sensitivity was similar for the two imaging methods (0.95). The increased specificity was mainly due to LC-OCT’s ability to differentiate basal cell carcinoma from other malignancies.

Two studies were devoted to the use of diffuse reflectance spectroscopy (DRS). V. Perekatova et al. reported on the comparative study of self-calibrating and single-slope DRS in terms of stability against various measurement disturbances [26]. The authors designed an experimental setup for DRS in a wide spectral range (from VIS to NIR), including a fiber-optic probe with two fibers for delivery and two used to receive light. This setup was capable of measuring with both single- and dual-slope (self-calibrating) approaches. The resistance of self-calibrating and traditional single-slope approaches to different instrumental disturbances was studied in phantom and *in vivo* experiments in human skin. The new method showed high stability to instrumental disturbances introduced into the source and detection channels, while the traditional single-slope approach demonstrated stability only to perturbations in the source channels.

In another study [27] A. Selifonov et al. used spectrophotometry measurements of diffuse reflectance and total transmittance in a wide spectral range (from 200 to 800 nm) to analyze the optical and molecular diffusion properties of cat ovarian tissues in the follicular and luteal phases using glycerol as an optical clearing agent. The authors found that the efficiency of optical clearing was significantly lower for the ovaries in the luteal phase compared with the follicular phase. The authors claim that the ability to recognize the phase in which the ovaries are stated, using their approach, could be useful in the context of cryopreservation, new reproductive technologies and ovarian implantation.

Finally, I. Zlotnikov et al. demonstrated the use of confocal laser scanning microscopy (CLSM) and Fourier-transform infrared spectroscopy (FTIR) to study the interactions of antibacterial drugs with bacterial cells [28]. More specifically, using these optical techniques, the authors studied the efflux effect of drug molecules from E.coli bacterial cells. It was demonstrated that eugenol, which acts as an adjuvant to rifampicin, significantly increased the penetration of the antibiotic and the maintenance of its intracellular concentration. In addition, optical methods were used to study bacteria localized inside macrophages, where the availability of bacteria for antibiotics is reduced, and an approach for effective drug delivery inside macrophages was demonstrated.

Thus, this Special Issue contains new developments and advanced ideas pertaining to optical spectroscopy and imaging for active translation to biological and clinical practice. The published manuscripts provide new insight into state-of-the-art modern methods of biomedical optics and expand readers’ knowledge of the possible areas of their application.

The peculiarity of biophotonics is that it combines physics, biology and medicine. The interdisciplinarity of biophotonics allows scientists from different fields to unite and collaborate to solve complex problems. Biophotonics and related fields are continuously developing and will undoubtedly become the main avenue for the development of biomedical engineering in the coming decades.
